# Immune modulation in response to coffee intake: a pilot study

**DOI:** 10.1007/s00394-026-03913-z

**Published:** 2026-02-16

**Authors:** M. Bleffgen, R. Lang, K. Rogal, V. Somoza, Thomas Skurk

**Affiliations:** 1 ZIEL Institute for Food and Health, Core Facility Human Studies, Technical University of Munich, Gregor-Mendel-Straße 2, 85354 Freising, Germany; 2 Leibniz Institute for Food Systems Biology, Technical University of Munich, Lise-Meitner-Straße 34, 85354 Freising, Germany; 3 Institute of Physiological Chemistry, Universität Wien, Josef-Holaubek-Platz 2, 1090 Vienna, Austria; 4 School of Medicine and Health, Klinikum rechts der Isar, Technical University of Munich, Ismaninger Straße 22, 81657 Munich, Germany

**Keywords:** Caffeine, Coffee brew, Immune system, Immune alertness

## Abstract

**Purpose:**

Coffee consumption has been associated with various health benefits; however, the underlying biological mechanisms remain poorly understood. In particular, the acute effects of coffee on circulating cytokines and the specific role of caffeine compared with the whole coffee matrix are still insufficiently characterised in humans. To our knowledge, no previous human study has directly compared immune modulation by coffee versus caffeine alone. We therefore aimed to elucidate the effect of a usual caffeine dose of 130 mg on postprandial cytokine secretion, and whether responses differ between coffee and pure caffeine.

**Methods:**

In a randomized pilot trial, ten healthy volunteers completed three test days receiving either a coffee brew, an aqueous caffeine solution (each 130 mg caffeine/100 ml), or water. Quantitative analysis of caffeine was performed with UHPLC-MS/MS, immune markers were measured by a multiplex immunoassay.

**Results:**

Our study demonstrates that both caffeine and coffee consumption influence the immune homeostasis, albeit with notable differences on cytokine secretion. Pure caffeine induced a higher anti-inflammatory response, as evidenced by the significant decrease in pro-inflammatory cytokines such as IL-17 A, IL-12p70, and IL-2 compared to coffee and water. The integrated response on the immune system is exemplified by the decrease of the pro-inflammatory IFN-γ (0.649 ± 0.068) and the anti-inflammatory IL-10 (0.478 ± 0.043), vs. baseline, respectively.

**Conclusion:**

These findings provide novel in vivo evidence that usual coffee and caffeine acutely affects cytokine responses differently in healthy individuals. In conclusion, our study addresses an important gap regarding the immune properties of coffee and suggests that bioactive compounds beyond caffeine contribute substantially to its immunological effects.

**Supplementary Information:**

The online version contains supplementary material available at 10.1007/s00394-026-03913-z.

## Introduction

Coffee is one of the most widely consumed beverages worldwide. It is known not only for its stimulating effects on cognitive and physical performance, but also for its potential health benefits [1, 2]. Epidemiological studies have linked regular coffee consumption to a reduced risk of chronic diseases, including type 2 diabetes [3–5] and cardiovascular disease [6–10]. While these associations are well documented [3, 4, 6–9], the underlying biological mechanisms remain incompletely understood. It is hypothesised that coffee’s bioactive compounds, particularly caffeine and antioxidants such as chlorogenic acid, play a role in modulating systemic inflammation and immune responses [4, 10–12].

Systemic inflammation is a key driver in the pathogenesis of many metabolic and cardiovascular diseases. Investigating the relationship between coffee consumption and immune biomarkers can provide valuable insights into how coffee influences inflammatory pathways. For example, Kempf et al. demonstrated that regular coffee consumption appears to have beneficial effects on subclinical inflammation [13]. While coffee contains various bioactive compounds, emerging evidence suggests that it is caffeine in particular that is responsible for many of its immunomodulatory effects [14, 15].

Caffeine’s immunomodulatory effects can be explained by two well-characterised mechanisms. Firstly, caffeine acts as an antagonist at adenosine receptors, particularly the A_2A_ receptor, which plays a pivotal role in immune regulation [14, 15]. Typically, adenosine signalling exerts immunosuppressive effects by downregulating pro-inflammatory cytokines such as TNF and IL-12, while enhancing anti-inflammatory cytokines such as IL-10 [16–19]. By inhibiting adenosine activation, caffeine may alter immune cell activity and, consequently inflammatory responses [20, 21]. Secondly, caffeine inhibits cyclic adenosine monophosphate–phosphodiesterase (cAMP-PDE), leading to elevated intracellular cAMP levels and the activation of protein kinase A (PKA) [14, 22]. This pathway is associated with the suppression of pro-inflammatory cytokine production, such as TNF and IL-12 and the promotion of anti-inflammatory mediators, like IL-10 [23–25]. Al Reef et al. proposed a mechanistic interplay between these pathways, noting that caffeine binding to the A_2A_ receptor increases cAMP levels by inhibiting PDE [15].

Numerous studies indicate that, like other methylxanthines, caffeine predominantly exerts anti-inflammatory effects. For example, it has been shown to suppress the migration of neutrophil and monocyte, while reducing the production of pro-inflammatory cytokines such as TNF in human blood samples [23, 26, 27]. Furthermore, caffeine has been linked to reduced T-cell proliferation and attenuated secretion of pro-inflammatory cytokines, including IL-2, IFN-γ, and IL-5 [14, 28].

Importantly, these effects have been observed at physiologically relevant caffeine concentrations, corresponding to habitual coffee consumption [14]. However, much of the existing evidence is derived from in vitro studies, highlighting the need for more in vivo research to establish the clinical and epidemiological significance of caffeine’s immunomodulatory properties [15].

To address this gap, we conducted a randomised, controlled pilot intervention investigating the effects of a typical 130 mg caffeine dose on postprandial cytokine secretion. We assessed a comprehensive panel of cytokines was assessed to evaluate immune balance. Additionally, we explored whether the form of caffeine administration (coffee brew versus aqueous caffeine solution) modulates cytokine responses differentially. By analysing cytokine dynamics following caffeine ingestion, we aimed to clarify how caffeine modulates immune function, both as a pure compound and as part of the coffee matrix. These findings could contribute to a deeper understanding of the potential roles of coffee and caffeine as functional food constituents or therapeutic agents in immune regulation.

## Materials and methods

### Human intervention study

Procedures for this pilot intervention study were followed in accordance with the ethical standards of the Helsinki Declaration, current Good Clinical Practice (ICH-GCP), and the local regulatory requirements. The study protocol was reviewed and approved by the ethics committee of the School of Medicine and Health Technical University of Munich, Germany (5798/13 S-SR). Before enrolment, each participant received written and oral explanations of the study procedures, potential risks, withdrawal rights and data confidentiality. Written informed consent was obtained by trained study stuff. The study was prospectivelyregistered in the German Clinical Trials Register (DRKS: DRKS00005083), including eligibility criteria, intervention details, and prespecified outcomes.

**Fig. 1:**
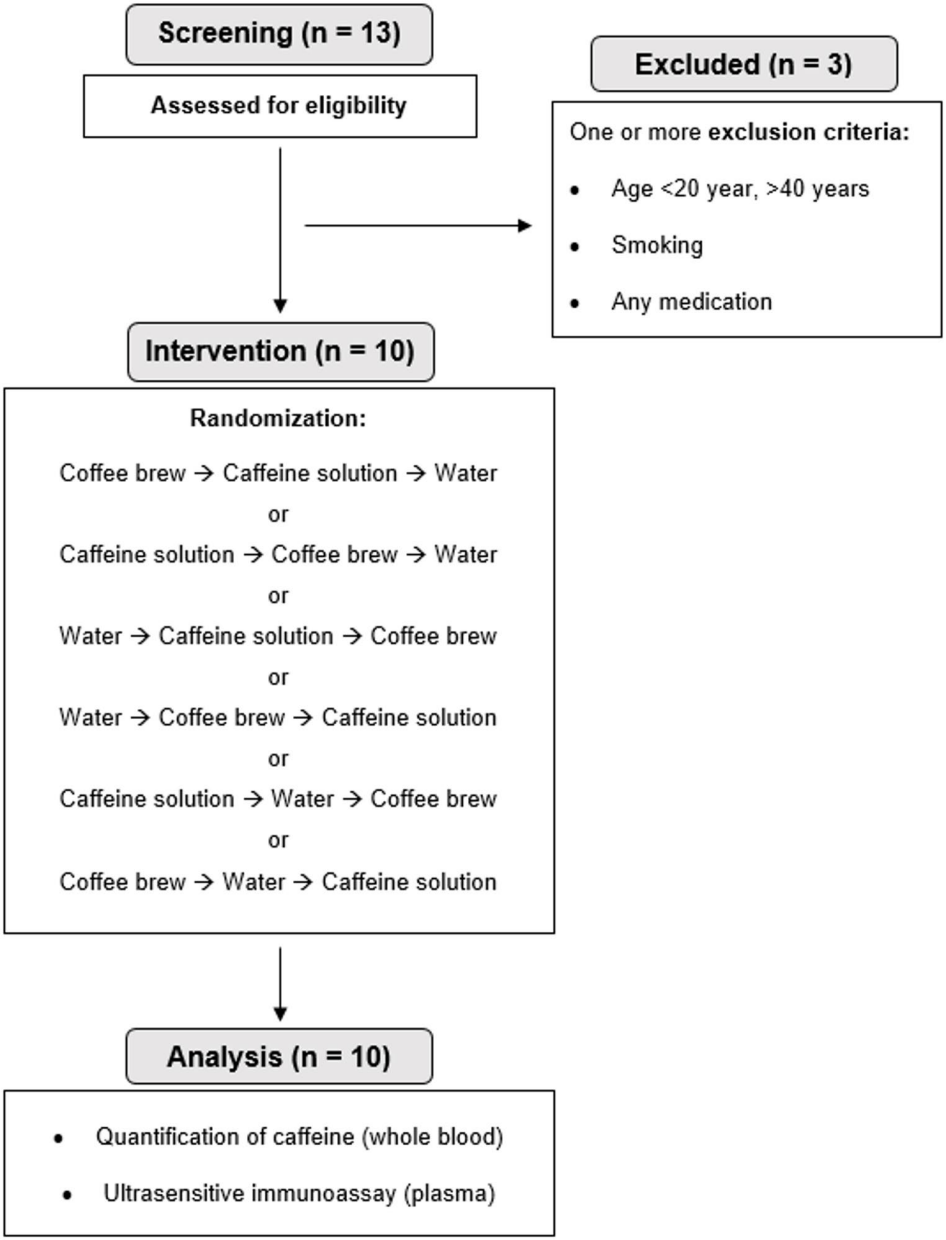
CONSORT participant flow chart

*Study participants.* Thirteen healthy volunteers were recruited via flyers at the Technical University of Munich, campus Freising. Enrolment of participants started on 5 November 2021, and continued until 17 February 2022. Participants’ eligibility was assessed with a detailed screening questionnaire. Exclusion criteria were age < 20 years and > 40 years, BMI > 25 kg/m^2^, smoking, taking any medication, practicing competitive sports and known metabolic disorders such as malabsorption syndromes, lipid metabolism disorders, diabetes mellitus, and thyroid dysfunction. To minimize interindividual variability in caffeine metabolism, tolerance, and sensory perception, only healthy individuals who regularly consumed coffee were included. This deliberate design choice enhanced the study´s internal validity and enabled more controlled interpretation of physiological responses to the intervention. Finally, ten participants were enrolled; Fig. 1 shows the participant flow chart of the study. Participants received financial compensation for their time, effort and travel expenses.

**Fig. 2:**
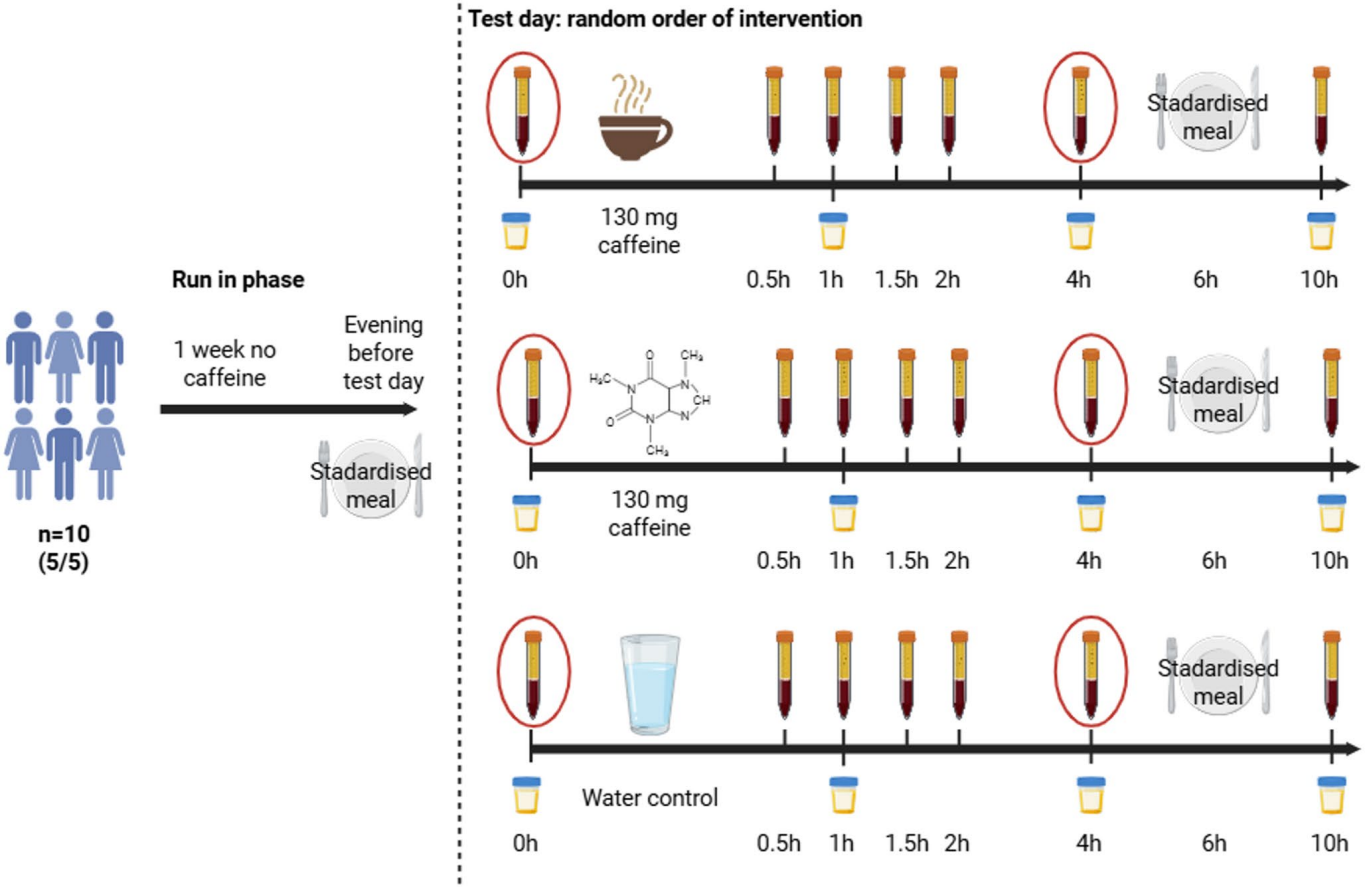
Study design of the pilot intervention: all participants (n = 10) underwent the coffee brew, caffeine solution and water intervention randomly. One week before the test day, it was prohibited to consume any caffeine-containing food. The evening before the test day, participants received our standardised meal. The same meal was provided for lunch on the test day, six hours after the intervention. Blood and urine were sampled at the time points indicated. The red-circled blood samples were analysed with immune assays. Created in BioRender. Haas, M. (2025) BioRender.com/u06y391

*Study design.* Ten healthy volunteers (5 females and 5 males) participated in the pilot study. Figure 2 depicts the study design of the intervention. Each participant underwent three test days, during which they consumed one of the following: coffee brew, an aqueous caffeine solution or a water control. Blinding of participants was not feasible in this study due to the distinct sensory differences (taste, appearance and smell) between the three interventions. To minimise potential bias, a randomised crossover design was employed. Each participant received all three interventions in a randomised order. Specifically, six intake sequences were generated: (1) Coffee/Caffeine/Control, (2) Coffee/Control/Caffeine, (3) Caffeine/Coffee/Control, (4) Caffeine/Control/Coffee, (5) Control/Coffee/Caffeine and (6) Control/Caffeine/Coffee. These sequences (1–6) were randomly assigned to participant IDs using the INDEX() and RANDBETWEEN(1,6) functions in Microsoft Excel. The participants completed a one-week washout phase before the three test days, during which caffeine-containing products were not permitted to reduce potential carryover effects from habitual caffeine intake. We provided an exclusion list detailing prohibited items, including coffee products, chocolate, black/green/white tea, mate tea, cola, energy drinks, and beauty products containing caffeine (e.g., shampoos and creams). Furthermore, the study participants received a standardised meal the evening before the intervention days. As this was an exploratory pilot study, no formal power calculation was conducted. Instead, the sample size was determined based on current recommendations for pilot studies in human nutrition research [29, 30]. Specifically, we followed the guidelines outlined by Ulaszewska et al. [30], which suggest that a sample size of approximately 10–12 participants is appropriate for pilot interventions aimed at assessing feasibility and logistics, and generating preliminary data. To further illustrate the adequacy of this approach, we additionally performed a post hoc power analysis (G*Power 3.1.9.7). Assuming a medium effect size (Cohen’s d = 0.53), α = 0.05, and a power of 0.8, the required sample size was estimated as 9 participants, which was met by our final sample of 10. However, we note, that such analyses are limited, and emphasise that the primary aim of our study was not to draw definitive conclusions, but rather to inform the design of future randomized controlled trials with adequate statistical power.

*Test day.* On the test days, participants were in a fasted state and received either a caffeine solution (130 mg caffeine dissolved in 100 ml water, standardised to the capsules), a 100 ml coffee brew made from two capsules (one capsule contained 65 mg caffeine; Nespresso^®^ ROMA, Nespresso Deutschland GmbH, Düsseldorf, Germany) or 100 ml water as a control. Adverse events were assessed based on participant self-report. At the end of each test day, participants were asked about whether they had experienced any discomfort, symptoms, or unusual reactions. All responses were documented.

*Blood and urine samples.* At the start of each test day, an intravenous catheter was inserted into an antecubital vein and remained in place until the final blood sample was collected. Blood samples were taken before coffee/caffeine/water intake (baseline = 0 h) and at defined time points (0.5, 1.0, 1.5, 2.0, 4.0, and 10.0 h) after the intervention. Six hours after consuming the beverage intake, the participants received the same standardised meal that they had eaten the previous evening. Urine samples were collected in the morning and at 1, 4, and 10 h. All samples were immediately aliquoted and stored at -80 °C until further analysis.

*Standardised meals.* The meal consisted of pasta (Barilla Spaghetti Nr. 5), butter, and salt (non-iodised). The pasta was cooked for 9 min and then mixed with butter and salt while still hot. The energy content of each meal was calculated based on the individual participant’s energy requirements (resting metabolic rate [RMR] x physical activity level [PAL]). One standardised meal corresponded to 1/3 of the daily energy requirement. The meal composition was based on 60% carbohydrates, 12% protein, and 28% fat. This equated to 76% pasta and 24% butter.

*Calculation of individual energy demand.* RMR was calculated based on gas exchange measurements performed by indirect calorimetry with the MetaLyser 3B-R3 from Cortex using a breathing mask (CORTEX Biophysik GmbH, Leipzig). Data were acquiredfor 30 min under thermoneutral conditions. To determine the individual energy demand, the RMR was multiplied with the participants individual PAL (1.2 for exclusively sedentary or reclining lifestyle, 1.4 for exclusively sedentary work with little or no strenuous leisure activity, 1.6 for sedentary work, occasionally with additional energy expenditure for walking or standing tasks, 1.8 for predominantly walking and standing work, 2.0 for physically demanding professional work) [31].

### Quantification of caffeine in human whole blood

*Chemicals.* Caffeine (99%) and ^13^C_3_-caffeine (solution, 1 mg/ml in methanol) were purchased from Merck KGaA (Taufkirchen, Germany). Porcine EDTA blood was obtained from previous studies [32].

*Standards*. A stock solution of caffeine was prepared in 20% aqueous ethanol at a final concentration of 500 µM. This solution was serially diluted in 1 + 1 steps with 20% aqueous ethanol to obtain concentrations of 250, 125, 62.5, 31.3, 15.6, and 7.8 µM. An aliquot (100 µl) of each dilution was added to matrix blood (porcine blood, 900 µl) to yield matrix standards with caffeine concentrations ranging from 0.78–50.0 µM. The methanolic ^13^C_3_-caffeine-solution (1 mg/ml, Merck KGaA, Taufkirchen, Germany) was diluted to a final concentration of 10 µM with 20% aqueous ethanol and served as the internal standard solution. The matrix samples were used to prepare a calibration curve for quantitation. Additional samples (25.0 and 1.56 µM) were prepared in triplicates (*n* = 3) and served as quality controls (QC).

*Sample preparation.* The matrix standard, QC samples and authentic blood samples from the human intervention study were prepared in a similar way. An aliquot (50 µl) was subsequently mixed with the internal standard solution (50 µl) and a mixture of acetonitrile/methanol (9/1, 300 µl). The resulting suspension was then centrifuged (4 °C, 12,500 rpm, 15 min) and an aliquot of the clear supernatant was subsequently evaporated. The residue was dissolved in water (200 µl).

*Calibration and quality controls (QC).* Calibration standards were analysed in triplicate. The calibration curves (y = mx+b, linear regression, 1/x weighing) was [Area(caffeine/13C3-caffeine)] = 1.19 × [concentration(caffeine/13C3-caffeine)] + 0.037 (R^2^ = 0.998). The back-calculated standards 1.56–50.0 µM fell between 80 and 120% accuracy (81.9–111.1%) and were < 13% relative standard deviation (0.2–12.1%). caffeine eluted at 3.00 min (± 4%).

Accuracy values for back-calculated calibration standards and QC samples are summarized in Supporting Table S1 and S2.

*Instrumental analysis*. The chromatographic system consisted of a Shimadzu Nexera X2 ultraperformance liquid chromatography (UPLC) system (Shimadzu, Duisburg, Germany), comprising an Autosampler (SIL 30AC, kept at 15 °C), two pumps (2 × LC-30AD), a degasser (DGU 20 A5R), a column oven (CTO 30 A, kept at 40 °C), and a communication device (CBM 20 A). The UPLC system was connected to an AB Sciex 5500 Qtrap mass spectrometer (Sciex, Darmstadt, Germany) operating in positive electrospray mode. Analyst 1.6.2 was used for instrument control and data analysis. The settings were as follows: Curtain Gas, 30; collision gas, “medium”; ion spray voltage, + 5.5 kV; source temperature, 550 °C; nebulizer gas, 60; and heater gas, 60. Resolution was set to “unit”. The dwell time for each mass transition was 50 ms with 5 ms between mass transitions. Declustering potential and entrance potential were 80 and 10. MRM-traces were as follows (CE, CXP, quantifier is marked with *): caffeine 194.9 > 138.0 (27, 18), 109.9 (31, 18), 83.0* (37, 16). ^13^C_3_ -caffeine 197.9 > 139.9 (25, 20), 111.9 (31, 18), 85.9 (39, 14) 69.9* (33, 10). The samples were separated on a Kinetex C18 column (1.7 μm, 100 × 2.1 mm, Phenomenex, Aschaffenburg, Germany) with 0.1% formic acid in water (eluent A) and 0.1% formic acid in acetonitrile (eluent B) at a flow rate of 400 µL/ min. After injection (1 µl), B was kept at 5% (1.5 min) and then increased from 5% to 70% in 1.5 min followed by 0.5 min of isocratic elution. The starting conditions were re-established within 0.2 min, and equilibration was 1.3 min prior to the next injection.

### Ultrasensitive immunoassay

We used the multi-array ultrasensitive MSD S-PLEX^®^ Proinflammatory Panel 1 (Human) assay kits (MSD, Rockville, MD, USA) to quantify a panel of nine pro- and anti-inflammatory cytokines (IFN-γ, IL-1β, IL-2, IL-4, IL-6, IL-10, IL-12p70, IL-17 A, TNF). The assay uses a sandwich immunoassay format with an electro-chemiluminescent (ECL) signal to detect plasma biomarker levels in the fg/mL range. The assay performed according to the manufacturer’s, using 25 µL of undiluted plasma or quality control (QC), and all samples were analysed in duplicated in side-by-side wells. We used the MESO QuickPlex SQ 120MM (with Methodical Mind software) to detect ELC signals. Overall, three MSD S-PLEX^®^ plates were measured, each including an 8-point calibration curve and QC samples in duplicate. Assay precision was determined by calculating the coefficient of variation (%CV) of intra-plate control replicates, yielding an average intra-assay CV of 7.58% across all plates. Consecutive data analysis was performed using MSD’s DISCOVERY WORKBENCH Version v4.0. Plasma samples collected at baseline (0 h) and after 4 h were analysed using this assay.

### Calculations and statistics

Blood caffeine concentrations were calculated using Analyst 1.6.3 (Sciex, Darmstadt, Germany). The data were then analysed and visualised using GraphPad 10.3.1.

**Fig. 3:**
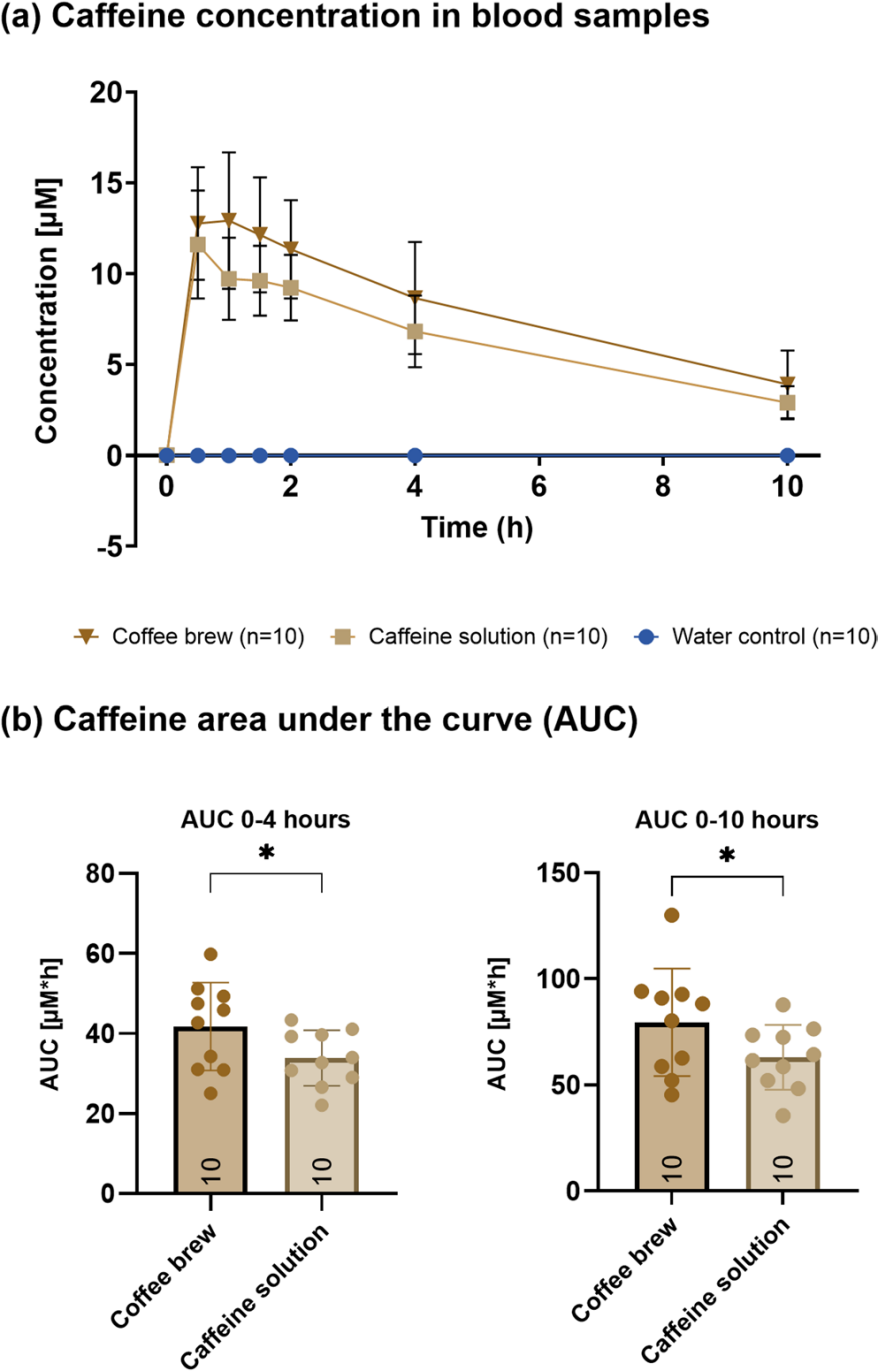
Panel **a** shows the plasma caffeine concentration [µM] over time (hours) for the three different interventions: water control (blue), coffee brew (yellow), and caffeine solution (brown). Data is presented as mean ± SD. Panel **b** compares the area under the curve (AUC) for caffeine concentrations between coffee brew and caffeine solution for 0–4 h and 0–10 h. The bars represent the mean ± SD and individual dots represent values for each participant. The asterisk (*p < 0.05) indicates significance between interventions. For both panels **a/b** in both panels three missing values (at 4 and 10 h) were interpolated using a first-order kinetic model. The model was applied using the mean values of all available data points, and the missing values were calculated at the corresponding time points based on their maximum concentrations

Unless otherwise stated, data in all graphs are represented as mean ± SD unless otherwise stated. For the plasma caffeine concentration plot (Fig. 3*)*, in both panels three missing values at 4 and 10 h were interpolated using a first-order kinetic model. This model was applied using the mean values of all available data points, with the missing values being interpolated at the corresponding time points based on their maximum concentrations.

Statistical significance was assessed using paired tests based on the normal distribution, as determined by the Shapiro-Wilk-test and QQ plots. For comparisons involving two groups, either a paired *t*-test or a Wilcoxon signed-rank test was applied. For comparisons involving more than two groups, one-way ANOVA with Bonferroni correction (parametric) or Friedman’s test with Dunn’s multiple comparisons (non-parametric) was used.

For postprandial cytokine release, the fold change from baseline to each measurement point was calculated and presented. An asterisk (*) indicates significance between interventions, and ahashtag (#) indicates significance between baseline and the respective 4-hour measurement.

## Results

### Study cohort characterization

Thirteen volunteers were screened based on the inclusion and exclusion criteria, and ten were finally enrolled. The final analysis dataset comprised 10 participants (5 females, 5 males). Baseline characteristics are shown in Table 1. All participants were regular coffee consumers. No adverse effects related to the interventions were observed.

**Table 1:** Study participant´s baseline characteristics

Number of participants, n	10
Female, n	5
Male, n	5
Age, mean ± SD (years)	28.3 ± 2.3
Weight, mean ± SD (kg)	69.31 ± 12.40
BMI, mean ± SD (kg/m^2^)	21.89 ± 2.22
Fat mass, mean ± SD (%)	21.60 ± 5.94
Fat free mass, mean ± SD (%)	78.40 ± 5.94
Muscle mass, mean ± SD (kg)	26.27 ± 6.54

### Postprandial blood caffeine concentration depends on method of caffeine administration

Figure 3a shows blood caffeine concentrations over time after following the consumption of coffee brew, caffeine solution and water. As expected, the water control intervention (blue) had no effect on blood caffeine concentrations. In contrast, both the coffee brew and caffeine solution increased blood caffeine concentrations, peaking around 1–2 h after consumption. Although both interventions revealed similar kinetic profiles, the mean caffeine concentrations were higher after coffee consumption than after the caffeine solution. After the peak, caffeine concentrations decreased gradually, remaining detectable for up to 10 h. Figure 3b shows the area under the curve (AUC) for the caffeine concentration over two different time intervals (0–4 h and 0–10 h) for both coffee brew (AUC_0−4_ = 41.76 ± 11.00 µM×h, AUC_0−10_ = 79.49 ± 25.33 µM×h) and caffeine solution (AUC_0−4_ = 33.85 ± 6.93 µM×h, AUC_0−10_ = 63.06 ± 15.29 µM×h). Total caffeine exposure, as indicated by the AUC, was higher for the coffee brew, with statistical significance (*p < 0.05) observed between groups for both the 0–4 h (*p = 0.018) and 0-10-hour intervals (*p = 0.037).

### Cytokine response varies by caffeine administration mode

**Fig. 4:**
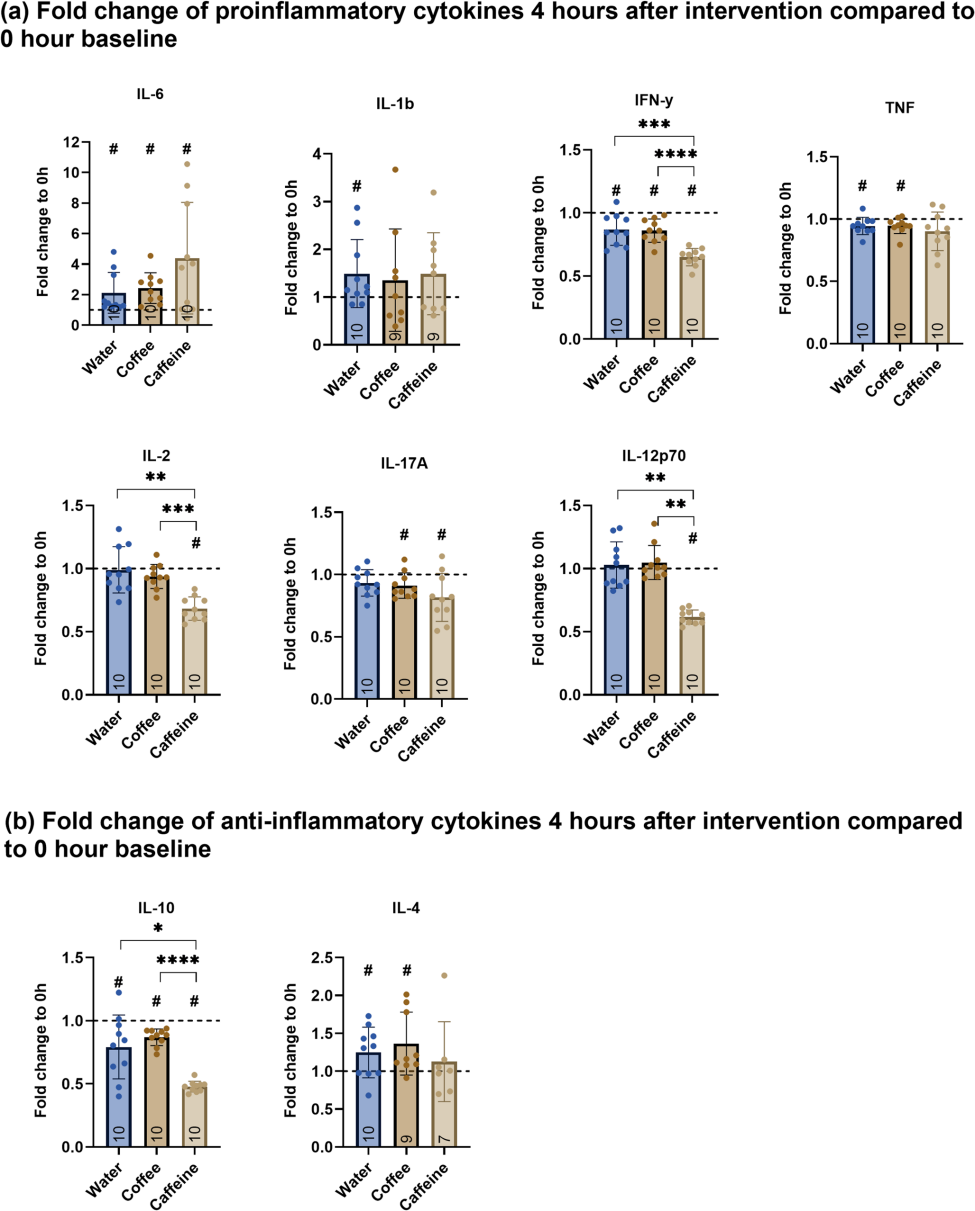
Comparison of postprandial cytokine secretion (fold change from baseline control at 0 h, represented by the horizontal line at y = 1) measured in plasma 4 h after ingesting 130 mg of caffeine, between coffee brew, caffeine solution, and water control. Panel **a** shows proinflammatory cytokines and panel **b** shows anti-inflammatory cytokines. The bars represent the mean ± SD and individual dots represent values for each participant. The number at the bottom of the bar indicates the number of participants. Hashtag (#) indicates significance (#*p* < 0.05) between baseline and respective intervention (water/coffee/caffeine) 4 h after consumption. Depending on normality distribution, either a paired *t*-test or Wilcoxon signed-rank test was used. Asterisks (*p < 0.05, **p < 0.01, ***p < 0.001, ****p < 0.0001) indicate significance between interventions (water/coffee/caffeine) 4 h after consumption. Depending on the normality distribution, either a one-way ANOVA with Bonferroni correction or Friedman’s test with Dunn’s multiple comparison was used. Due to missing data (NAs) Kruskal-Wallis-test was applied for IL-1β and IL-4

The results indicate heterogeneous cytokine secretion following interventions with coffee brew, caffeine solution, and water control. Figure 4 illustrates the fold change (FC) in cytokine levels 4 h after ingestion, relative to baseline levels (0 h). Panel (a) displays proinflammatory cytokines (IFN-γ, IL-1β, IL-2, IL-6, IL-12p70, IL-17 A, TNF), and panel (b) shows the anti-inflammatory cytokines (IL-4, IL-10).

Coffee consumption closely mirrors the water control for both pro- and anti-inflammatory cytokine release. Although significant deviations from baseline were observed for IL-6, IFN-γ, TNF, IL-4, and IL-10 following coffee brew and water interventions (#*p* < 0.05), no significant differences were found between the two groups.

In contrast, caffeine substantially suppressed both pro- and anti-inflammatory cytokines, particularly IFN-γ, IL-12p70, IL-2, and IL-10, as indicated by a marked (#*p* < 0.05) decrease in FC relative to baseline (IFN-γ = 0.649 ± 0.068, IL-12p70 = 0.616 ± 0.057, IL-2 = 0.683 ± 0.092, IL-10 = 0.478 ± 0.043) and in comparison to both the water control and coffee brew. For other cytokines, such as IL-6, IL-1 β IL-1β, and IL-4, levels increased, while TNF and IL-17 A decreased from baseline (#*p* < 0.05).However, no significant differences were observed between the three groups. Since water served as the control, changes that deviate from those observed in the water group should be noted, as they highlight caffeine’s distinct immunomodulatory effects. Explicit *p*-values can be found in Table 2.

**Table 2:** Explicit *p*-values for each cytokine

	*P*-values to respective baseline (0 h)	*P*-values from multiple comparison		
		Water to coffee	Water to caffeine	Coffee to caffeine
*IL-6*				
Water	**0.002**	< 0.999	*0.116*	
Coffee	**0.002**			*0.211*
Caffeine	**0.017**			
*IL-1β*				
Water	**0.048**	*0.915*	< 0.999	
Coffee	*0.347*			< 0.999
Caffeine	*0.126*			
*IFN-γ*				
Water	**0.008**	< 0.999	**< 0.001**	
Coffee	**0.001**			**< 0.001**
Caffeine	**< 0.001**			
*TNF*				
Water	**0.027**	< 0.999	< 0.999	
Coffee	**0.026**			< 0.999
Caffeine	0.077			
*IL-2*				
Water	*0.865*	< 0.999	**0.006**	
Coffee	*0.070*			**< 0.001**
Caffeine	**< 0.001**			
*IL-17 A*				
Water	0.077	< 0.999	*0.215*	
Coffee	**0.023**			0.405
Caffeine	**0.0146**			
*IL-12p70*				
Water	*0.642*	< 0.999	**< 0.001**	
Coffee	*0.625*			**< 0.001**
Caffeine	**< 0.001**			
*IL-10*				
Water	**0.028**	*0.903*	**0.001**	
Coffee	**< 0.001**			**0.001**
Caffeine	**< 0.001**			
*IL-4*				
Water	**0.043**	< 0.999	*0.940*	
Coffee	***0.012***			*0.380*
Caffeine	0.938			

*P*-values < 0.05 (**in bold**) were considered statistically significant. Significant *p*-values are written in bold. Comparisons between baseline (0 h) and the respective interventions (water, coffee, or caffeine) 4 h after consumption were performed using either a paired t-test or a Wilcoxon signed-rank test, depending on data normality. Comparisons between the three interventions at 4 h were conducted using either one-way ANOVA with bonferroni correction or friedman’s test with dunn’s multiple comparisons, as appropriate. Due to missing data (NAs), the Kruskal–Wallis test was applied for IL-1β and IL-4

## Discussion

Our study is the first to investigate in vivo postprandial cytokine release and blood caffeine kinetics following the consumption of coffee versus pure caffeine. Despite the identical caffeine doses (130 mg), the mode of administration affected both caffeine pharmacokinetics and immunomodulatory responses differently.

Our results show that blood caffeine concentrations were higher after coffee consumption than after the caffeine solution. This finding is counterintuitive, as it is generally assumed that caffeine in aqueous solutions is absorbed better, leading to higher peak plasma concentrations than caffeine from coffee or tea [33, 34]. However, there is a notable lack of studies directly comparing the pharmacokinetics of caffeine following the ingestion of pure caffeine and coffee drinks. We hypothesise that the complex composition of coffee may influence caffeine absorption, possibly through synergistic interactions with other coffee constituents that modify bioavailability. Additionally, variation in caffeine extraction during coffee preparation may have contributed to this finding, as opposed to the precisely measured caffeine in the aqueous solution.

Our findings confirm that caffeine significantly modulates immune function by suppressing both proinflammatory (IFN-γ, IL-12p70 and IL-2) and anti-inflammatory (IL-10) cytokines, when compared to both water control and coffee brew. Notably, IL-6 levels increased significantly across all interventions, with the most pronounced effect observed after caffeine ingestion. Given that IL-6 plays a dual role in inflammation and tissue repair [35, 36], its elevation may be partially attributable to mechanical factors, such as the body´s response to the intravenous catheter, rather than caffeine alone. Interestingly, the cytokine changes observed after coffee consumption closely resembled those from water control, suggesting that coffee´s immunomodulatory profile is more integrated than that of pure caffeine. By analysing cytokine levels at 4 h post-ingestion, we ensured that the observed effects were solely attributable to coffee or caffeine, excluding interference from food intake. This is consistent with our previous findings, which showed that later time points were necessary to reflect the induction of the transcriptional machinery in response to these nutrition compounds [37].

Our findings align with previous research demonstrating that caffeine suppresses cytokine production [23, 24, 26–28]. Notably, reductions in pro-inflammatory markers such as TNF, IFN-γ, and IL-12 have been reported consistently in numerous in vitro studies, as summarised in two major reviews [14, 15]. In our in vivo study, we observed comparable decreases in IFN-γ and IL-12p70, supporting the hypothesis that caffeine primarily modulates immune function through the inhibition of PDE [22]. Although the antagonistic effect on adenosine receptors would typically promote a pro-inflammatory response, our results suggest that caffeine has a strong overall anti-inflammatory effect [14]. Horrigan et al. recently demonstrated that 100 µM of caffeine solutions significantly increased intracellular cAMP levels by up to 120% in LPS-stimulated human monocytes which was linked to the suppression of TNF production [23]. However, the extent to which PDE inhibition contributes to immune regulation in humans remains unclear, as research on the effects of caffeine in immune cells is limited. Additional mechanisms, such as histone deacetylation [38] and adenosine A_3_ receptor activation [39], may also be involved. The decrease in IL-10 observed in our study suggests an alternative immunoregulatory pathway that is potentially mediated by adenosine receptor interactions.

Interestingly, despite matching the coffee and the aqueous caffeine solution for caffeine content, we observed higher plasma caffeine concentrations following coffee consumption. This counterintuitive result suggests the presence of potential matrix effects and interindividual differences in absorption or metabolism. Coffee is a chemically complex beverage containing polyphenols such as chlorogenic acids, and diterpenes, such as cafestol and kahweol. These compounds may influence gastrointestinal function, intestinal permeability, or hepatic enzyme activity, such as CYP1A2 [40, 41]. Preclinical studies in rats have shown that cafestol and kahweol can downregulate CYP1A2 mRNA expression [42], potentially reducing caffeine clearance and thereby increasing systemic exposure. Conversely, the aqueous caffeine solution lacks such matrix complexity, which may lead to differences in dissolution, absorption kinetics, or first-pass metabolism. Although both preparations were standardised for caffeine content, minor variations in extraction efficiency or compound stability during preparation cannot be entirely ruled out. Overall, the coffee matrix is more likely to modulate gastric function (stimulating secretion without accelerating gastric emptying), as well as possibly modulating intestinal transit and first-pass metabolism, thereby shifting early absorption and increasing systemic exposure relative to an aqueous caffeine solution [41]. These findings highlight the importance of considering food matrix interactions when interpreting the pharmacokinetics of bioactive dietary compounds [40–42].

As previously mentioned, coffee is a complex matrix of antioxidants and phytochemicals, many of which have been linked to various health benefits [4, 8, 14]. While caffeine is often discussed as the primary agent responsible for coffee’s immunomodulatory effects [14, 15], its activity may be significantly influenced by interactions with other compounds in the coffee matrix, most notably, chlorogenic acids. Chlorogenic acids, a major class of polyphenols, have demonstrated anti-inflammatory properties. These include downregulating of pro-inflammatory cytokines such as TNF-α and IL-6, inhibition of the NF-κB signalling pathway, and reduction of oxidative stress by enhancing endogenous antioxidant defences [11, 12]. These mechanisms may help to counteract modulating the immunoactive effects observed with the intake of isolated caffeine. Our results suggest that coffee consumption leads to a more nuanced cytokine response than caffeine alone. This interaction highlights the importance of considering coffee´s full phytochemical matrix of when evaluating its physiological impact, rather than attributing effects solely to caffeine content.

The observed modulation of cytokine levels *may* be of potential relevance in the context of low-grade inflammation, which is commonly associated with conditions such as obesity, type 2 diabetes, and cardiovascular disease. While our study was conducted in healthy individuals, and no direct conclusions can be drawn for clinical populations, however prior findings [3–5, 10] suggest that coffee might influence inflammatory pathways. This raises the possibility that long-term regular coffee consumption could contribute to the dietary prevention or modulation of chronic low-grade inflammation though further research is needed to explore this in a risk or patient populations.

A limitation of our study was the lack of analysis of various caffeine metabolites, which would have provided a more comprehensive insight into the interactions between coffee bioactives and cytokine release. As there is currently no published data specifically examining the relationship between cytokine levels in response to a controlled intake of coffee and caffeine, we designed an exploratory pilot study to investigate this association. Given the pilot nature of our study, the sample size was limited, potentially restricting generalisability. Generalisability is also skewed because we only included healthy, young coffee drinkers. As the study investigated the acute immune response, it was clearly limited in the interpretation of terms of long-term effects. However, the study design has significant advantages, as each participant served as their own control, minimising inter-individual variability with acquisition of dependent data. Furthermore, this study is among the first to investigate both, pro- and anti-inflammatory cytokines in response to coffee and caffeine intake, offering a more comprehensive view of immune regulation. Future studies should focus more specifically on dissecting matrix-related effects and include a decaffeinated coffee control to help distinguish between caffeine-dependent and caffeine-independent mechanisms. However, given that decaffeination processes can alter the overall coffee matrix and that decaffeinated brews are not entirely caffeine-free, this control must be carefully selected and interpreted.

## Conclusion

This is the first study to investigate postprandial cytokine release following caffeine/coffee consumption in vivo. Despite the identical caffeine doses, we observed significant differences in blood caffeine kinetics and cytokine profiles between the two administration methods. Pure caffeine induced a more pronounced anti-inflammatory pattern, characterised by reductions in several pro-inflammatory cytokines, whereas coffee intake led to a more integrated immunomodulatory profile involving increases in the anti-inflammatory cytokine IL-4 and attenuated pro-inflammatory markers. These findings suggest that coffee’s complex matrix, which is rich in bioactive compounds such as chlorogenic acids, may contribute to the modulatory effects of caffeine on the immune system differentiating it from pure caffeine.

While our results provide novel in vivo evidence that usual coffee and caffeine intake have differential acute effects on cytokine responses in healthy humans, these results should be interpreted in light of the small sample size, pilot study, and restriction to young, healthy participants under controlled conditions. Further studies with larger study cohorts are needed to confirm these observations, identify the specific compounds responsible for the differential effects, and explore their potential relevance in populations with chronic low-grade inflammation. Overall, our results emphasise the importance of considering the effects of the food matrix in nutritional immunology, and they support the continued investigation of coffee as a complex dietary factor with potential immunomodulatory properties.

## Supplementary Information

Below is the link to the electronic supplementary material.

Supplementary Material 1

## Data Availability

The data and materials that support the findings of this study are available from the corresponding author upon reasonable request.
