# Supplementary material for: Immune modulation in response to coffee intake: a pilot study
Source: Eur J Nutr. 2026 Feb 16;65(2):61. doi: 10.1007/s00394-026-03913-z (PMC12909337; doi:10.1007/s00394-026-03913-z)
Supplement: Supplementary file 1 — Supplementary Material 1 [file 394_2026_3913_MOESM1_ESM.docx]

**Supplemental Material**

Table S1. Accuracy and precision of back-calculated matrix standards (n=3).

| **Nominal (µM)** | **Found (µM)** | **Accuracy (%)** | **Precision (RSD, %)** |
| --- | --- | --- | --- |
| 0.78 | 0.64±0.08 | 81.9 | 12.1 |
| 1.56 | 1.73±0.09 | 111.1 | 5.3 |
| 3.13 | 3.05±0.07 | 97.6 | 0.2 |
| 6.25 | 6.62±0.15 | 105.9 | 2.3 |
| 12.5 | 13.08±0.35 | 104.7 | 2.7 |
| 25.0 | 25.26±0.85 | 101.1 | 3.4 |
| 50.0 | 48.82±1.13 | 97.6 | 2.3 |
|  |  |  |  |

Table S2. Accuracy and precision of QC samples (n=3).

| **Nominal (µM)** | **Found (µM)** | **Accuracy (%)** | **Precision (RSD, %)** |
| --- | --- | --- | --- |
| 1.56 | 1.45±0.03 | 92.9 | 2.4 |
| 25.0 | 25.40±0.35 | 101.6 | 1.4 |
|  |  |  |  |

Figure S1: Matrix-calibration curve for quantification of caffeine in blood. Area ratios (Analytes/IS) are plotted versus the analyte concentration. R2=0.9987, slope y=0.1146x+0.0022.





Figure S2: Accuracy data of quality controls (15.6 and 25.0 µM in porcine EDTA blood, n=3 each). Accuracy is given as (calculated concentration/nominal concentration) x100%.
